# MicroRNA-34c acts as a bidirectional switch in the maturation of insulin-producing cells derived from mesenchymal stem cells

**DOI:** 10.18632/oncotarget.21883

**Published:** 2017-10-16

**Authors:** Chunyu Bai, Yuhua Gao, Xiangyang Zhang, Wancai Yang, Weijun Guan

**Affiliations:** ^1^ Key Laboratory of Precision Oncology of Shandong Higher Education, Institute of Precision Medicine, Jining Medical University, Jining, 272067, PR China; ^2^ College of Basic Medicine, Jining Medical University, Jining, 272067, PR China; ^3^ Institute of Animal Sciences, Chinese Academy of Agricultural Sciences, Beijing 100193, PR China; ^4^ Department of Pathology, University of Illinois at Chicago, Chicago, IL 60612, USA

**Keywords:** insulin-producing cell, mesenchymal stem cells, differentiation, miR-34c, insulin secretion

## Abstract

miRNAs regulate insulin secretion, pancreatic development, and beta-cell differentiation. However, their function in the differentiation of IPCs from MSCs is poorly understood. In this study, to screen for miRNAs and their targets that function during the formation of IPCs from MSCs, we examined the miRNA expression profiles of MSCs and IPCs using RNA-seq and qPCR to confirm the above results. We found that miR-34c exhibited transient upregulation at an early stage of the formation of IPCs derived from MSCs. Next, we analyzed the biological function of miR-34c by predicting its targets using bioinformatic tools. Combining our data with those from previous reports, we found that miR-34c and its targets play an important role in the formation of IPCs. Therefore, we overexpressed miR-34c and expressed small interfering RNAs of its targets in MSCs to investigate their functions in IPC formation. We found that miR-34c acts as a bidirectional switch in the formation of IPCs derived from MSCs by regulating the expression of targets to affect insulin synthesis and secretion. miR-34c was shown to downregulate its targets, including PDE7B, PDGFRA, and MAP2K1, to increase proinsulin synthesis, but when miR-34c continually dysregulated such expression, it suppressed the expression of other targets, namely ACSL4 and SAR1A, weakening insulin secretion in IPCs. These results suggest that endogenous miRNAs involved in the formation of IPCs from stem cells should be considered in the development of effective cell transplant therapy for diabetes.

## INTRODUCTION

Type I diabetes mellitus is caused by the autoimmune destruction of beta cells in pancreatic islets. While the replacement of insulin-producing cells would be the ideal solution for this disease [1], the generation of insulin-secreting cells from stem cells or other cells represents an attractive alternative [2, 3]. Mesenchymal stem cells (MSCs) are ideal donor cells for cell transplantation, as they possess a strong immunoregulatory effect and are multipotent. Umbilical cord-derived MSCs possess two critical advantages over other MSCs [4]. First, umbilical cord tissue is routinely discarded, so it is readily available for cord stem cell extraction. Second, the process of collecting MSCs from the umbilical cord is not invasive, so there is no risk to donors.

Genome-encoded miRNAs regulate gene expression post-transcriptionally [5]. A number of miRNAs are important regulators of beta-cell differentiation and function, including miR-375 [6], miR-26 [7, 8], miR-24, miR-148 [8], miR-200, miR-30d, miR-124a [9], miR-204 [10], miR-223, miR-21 [11, 12], let-7 [13, 14], miR-9, miR-15a, miR-16, miR-146a, miR-29a, and miR-34 [15, 16]. p53, a tumor suppressor, is tightly connected with canonical Wnt signaling and the epithelial–mesenchymal transition program through the miR-34 family [17], demonstrating that miR-34 directly targets the untranslated regions of a set of Wnt and EMT genes, including Wnt1, Wnt3, LRP6, AXIN2, β-catenin, LEF1, and Snail [18, 19]. The miR-34 family seems to be critically important in this axis because the depletion of endogenous miR-34a mostly abolished p53-mediated suppression of Wnt transcriptional activity. miR-34 includes three forms, miR-34a, miR-34b, and miR-34c, which are encoded at two distinct loci, mir-34a and mir-34b/34c. This miRNA is a transcriptional regulator of beta-cell function, and its expression is upregulated by exposure to proinflammatory cytokines or in prediabetic (nonobese diabetic) mice. However, the function of miR-34c in pancreatic beta-cell differentiation has not been fully investigated. To better understand the role of miRNAs in the differentiation of insulin-producing cells (IPCs) from MSCs, in this study we used RNA-seq analysis to characterize differences in miRNA expression profiles between IPCs and MSCs, and to confirm the significant change of miRNA during the differentiation of IPCs using qPCR. Combining our data with those from previous reports, we found that miR-34c and its targets play a critical role in the formation and maturation of IPCs. To elucidate the mechanism of transcriptional regulation during the differentiation of IPCs and to better understand the role of miR-34c in the maturation of IPCs from MSCs, we overexpressed or knocked down miR-34c and its target genes *in vitro*.

## RESULTS

### miR-34c signature in differentiation of IPCs derived from MSCs

**Figure 1:**
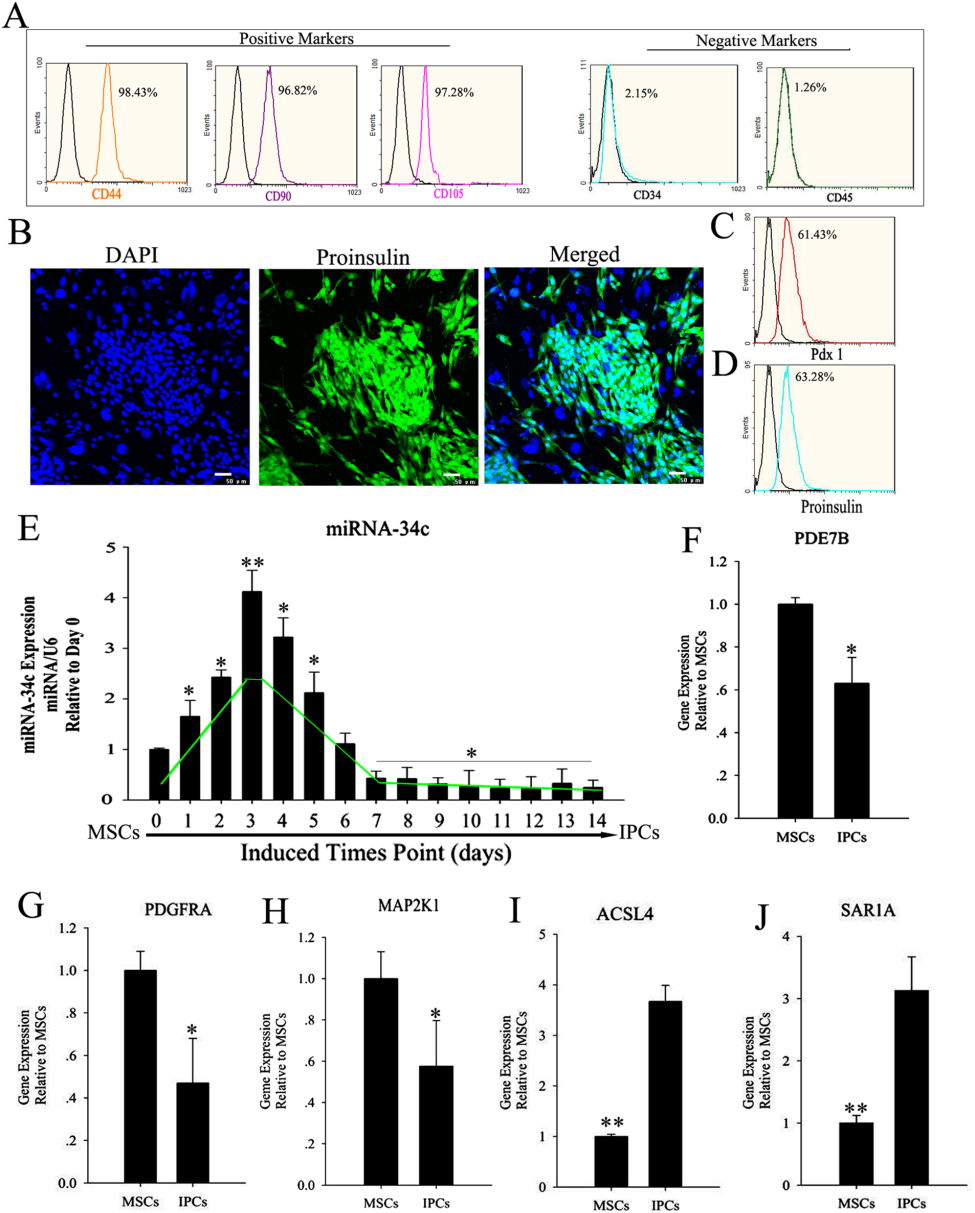
Expression level of miR-34c and its targets during the induction of MSC differentiation into IPCs **(A)** MSC surface markers were detected by flow cytometry. CD44, CD90, and CD105 positivity is a marker of MSCs, as is CD34 and CD45 negativity. Obtained MSCs express CD44, CD90, and CD105, but not CD34 and CD45. **(B–D)** MSCs were induced to differentiate into IPCs using cocktail factors. Following this treatment, MSCs adopted a round or ovoid morphology resembling that of pancreatic islet cells. Expression of the islet markers proinsulin and pdx1, as determined by immunofluorescence and flow cytometry. **(E)** Relative expression levels of miR-34c during the formation of IPCs; data were normalized to normal MSCs (day 0). The x-axis shows days after induction. **(F–J)** Relative expression of miR-34c targets in IPCs differentiated from MSCs, as determined by real-time polymerase chain reaction (PCR) analysis. Results are mean ± SEM of three independent experiments (^*^: p<0.05, ^**^: p<0.01).

MSCs are multipotent stem cells, which can differentiate *in vitro* into many cell types that express specific markers. Here, specific markers of MSCs were detected via flow cytometry assays. MSCs were positive for CD44, CD90, and CD105, and negative for CD34 and CD45 (Figure 1). Multi-differentiation potential is a special characteristic of MSCs, which can differentiate into cells of the three germ layers. We induced the differentiation of MSCs into insulin-producing cells (IPCs) using cocktail factors. We then observed changes in the transcription of the islet hormone genes Pdx1 and proinsulin using flow cytometry and immunofluorescence. miRNAs in animals exhibit tissue-specific or developmental-stage-specific expression, indicating that they play important roles in many biological processes [24, 25]. In this research, we applied RNA-seq technology to reveal the differential expression of miRNAs in MSCs and IPCs. The differential expression of miRNAs was classified into three groups according to their expression levels in the two cell types. Groups I and III contained 107 and 54 miRNAs, respectively. Group I consisted of miRNAs whose expression was upregulated (*p*<0.05), while group III contained miRNAs whose expression was downregulated in insulin-producing cells compared with that in MSCs. Group II comprised 250 miRNAs whose expression was relatively unchanged (*p* > 0.05) (Supplementary Table 1). To validate the RNA-seq results, the miRNAs related to pancreatic development were selected and their expression levels during the differentiation of IPCs were determined by relative quantification (RQ) using real-time PCR. RQ values were normalized against U6 as an endogenous control. We identified an interesting phenomenon in the RQ results, namely, that miR-34c showed transient upregulation during the formation of IPCs derived from MSCs. Then, the targets of miR-34c were predicted and analyzed in IPCs using the online tools TargetScan, miRDB, and miRbase. In combination with data from a previous study, ACSL4 (acyl-CoA synthetase long-chain family member 4), PDE7B (phosphodiesterase 7B), PDGFRA (platelet-derived growth factor receptor alpha), MAP2K1(mitogen-activated protein kinase kinase 1), and SAR1A (secretion-associated Ras-related GTPase 1A) were selected as candidate targets in this research. The relative target gene expression was compared between MSCs and differentiated IPCs.

### Identification of miRNA targets

**Figure 2:**
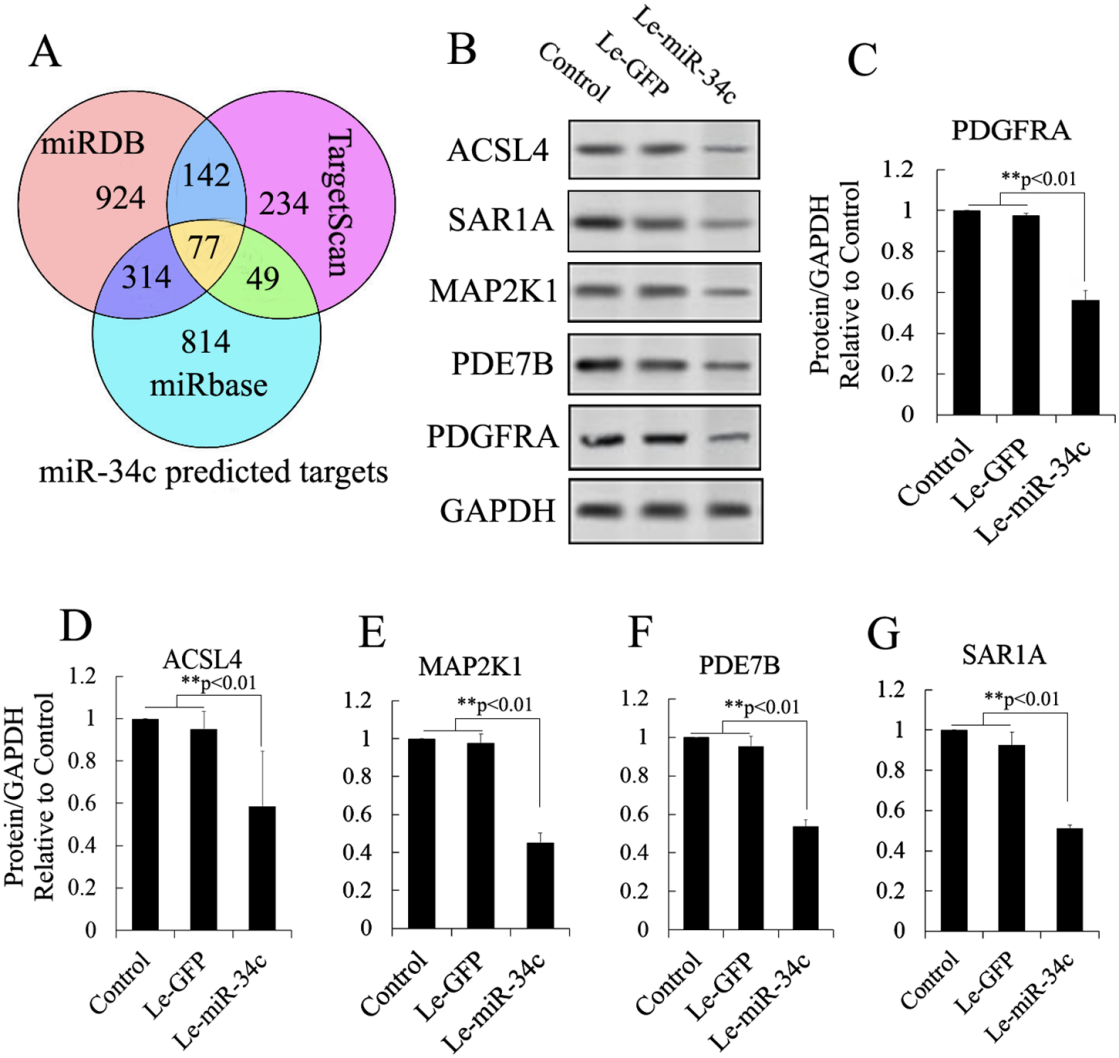
Candidate target analysis of miR-34c **(A)** Targets of miR-34c were predicted using TargetScan, miRDB, and miRBase tools, and analyzed using Venny tools. Seventy-seven direct target genes of miR-34c that are involved in the formation and maturation of IPCs were found in the Venn diagram. **(B)** Combining the findings with previous reports, ACSL4, PDE7B, PDGFRA, MAP2K1, and SAR1A were selected to test the regulatory relationship after miR-34c overexpression using western blotting. **(C–G)** Western blot analysis of proteins expressed by gene targets of miR-34c following miR-34c overexpression in MSCs. Protein abundance was analyzed using ImageJ tools. Overexpression of miR-34c for 72 h inhibited endogenous expression of ACSL4, PDE7B, PDGFRA, MAP2K1, and SAR1A (n = 5; paired two-tailed *t*-test, ^*^: p<0.05, ^**^: p<0.01). GAPDH was used as an endogenous control. Le-eGFP, lentivirus expressing enhanced green fluorescent protein (vector control); Le-miR-34c, lentivirus expressing miR-34c.

**Figure 3:**
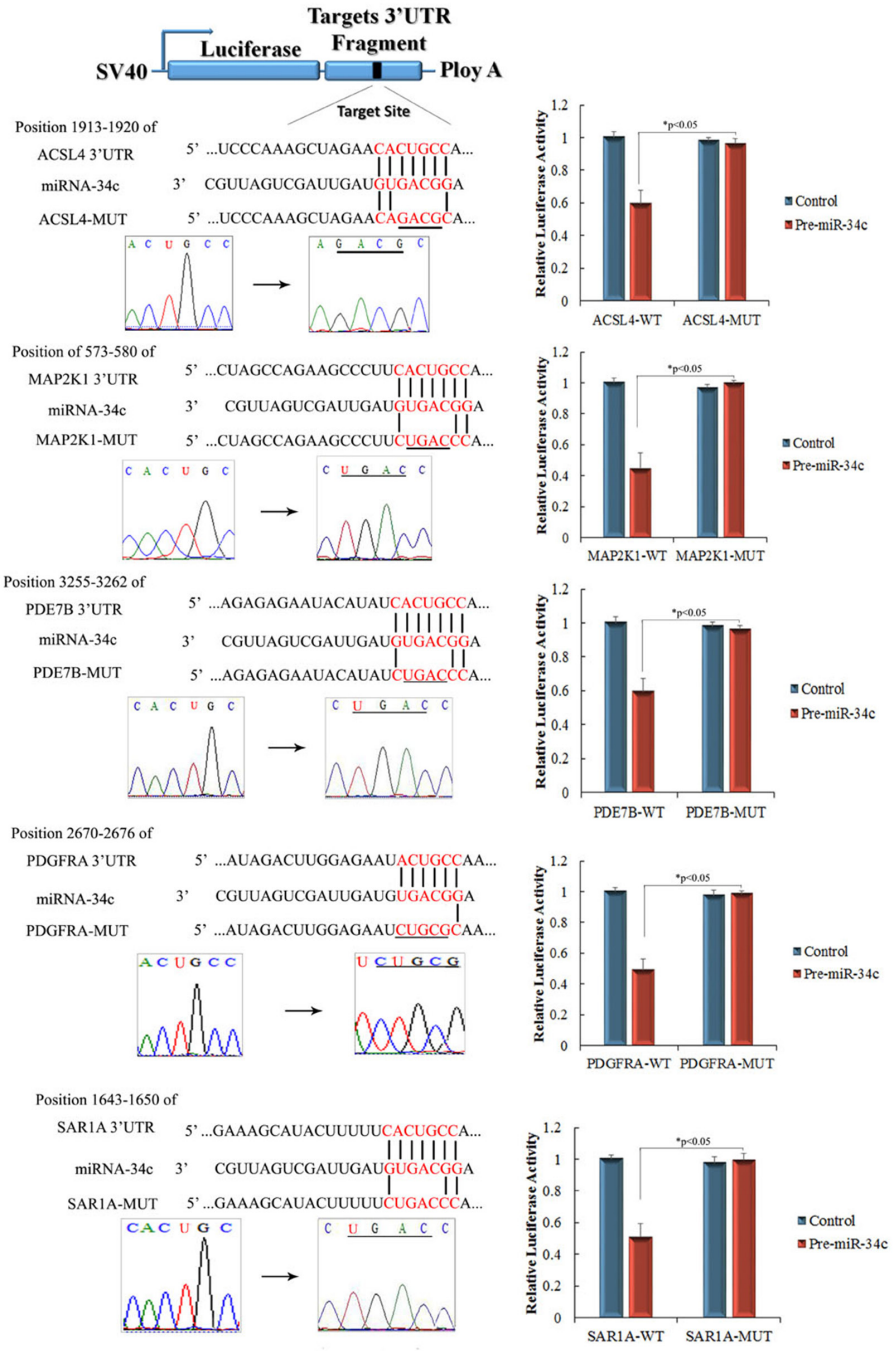
Identification of miR-34c targets using luciferase activity assays Predicted base pairing of mature miRNA sequences and 3′-untranslated regions (UTRs) of target genes. Seed sequences are shown in red. Complementary sites of miR-34c and the 3′-UTRs of ACSL4, PDE7B, PDGFRA, MAP2K1, and SAR1A are connected by vertical lines. The mutant sequences (ACSL4-MUT, PDE7B-MUT, PDGFRA-MUT, MAP2K1-MUT, and SAR1A-MUT) are identical to the wild-type constructs (ACSL4-WT, PDE7B-WT, PDGFRA-WT, MAP2K1-WT, and SAR1A-WT), except for four point mutations that disrupt base pairing at the 5′ end of miR-34c (underlined). Mutating the miR-34c target site in the 3′-UTR of ACSL4, PDE7B, PDGFRA, MAP2K1, or SAR1A abolished the inhibition of luciferase activity by endogenous miR-34c in 293T cells (paired two-tailed *t*-test, ^*^: p<0.05, ^**^: p<0.01).

**Figure 4:**
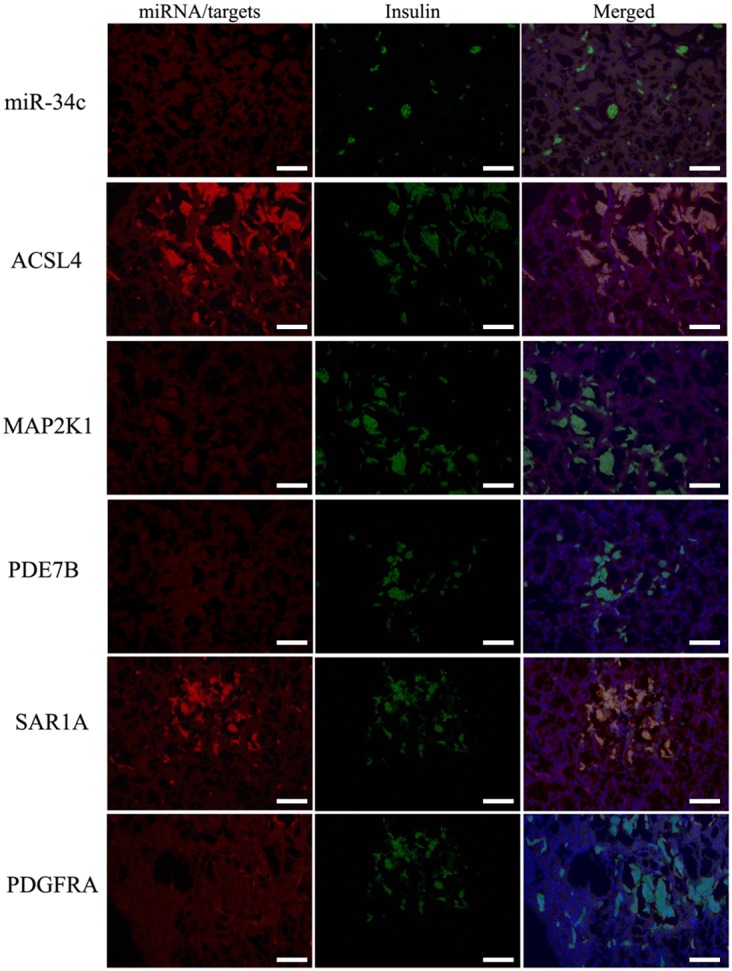
Expression of miR-34c and its targets in pancreatic tissue using *in situ* hybridization and IHC It has been demonstrated that miR-34c could directly regulate the expression of its targets ACSL4, PDE7B, PDGFRA, MAP2K1, and SAR1A *in vitro*, but no reports about the coexpression of miR-34c and its targets in normal pancreatic islets have been published. The results demonstrated that insulin is coexpressed with ACSL4 and SAR1A in pancreatic islets, but not with miR-34c, MAP2K1, PDE7B, and PDGFRA. The results suggested that miR-34c plays a critical role in maintaining the normal physiological function of beta cells (scale bar = 100 μm).

miR-34c target genes involved in the formation and maturation of IPCs were predicted and analyzed using TargetScan, miRDB, and miRBase (predicted target genes of miR-34c are listed in Supplementary Table 2. Potential target genes of miR-34c during the formation and maturation of IPCs were further identified using western blotting after the transfection of MSCs with miR-34c. Seventy-seven direct target genes of miR-34c that are involved in the formation and maturation of IPCs were found and are listed in Supplementary Table 3. ACSL4, PDE7B, PDGFRA, MAP2K1, and SAR1A were listed as potential targets of miR-34c, the targeting of which can influence the function of pancreatic beta cells [26–32]. Thus, to test the functions of miR-34c in MSCs, the precursors of miR-34c were overexpressed in MSCs. The expression of proteins encoded by the target genes, including ACSL4, PDE7B, PDGFRA, MAP2K1, and SAR1A, was found to be decreased relative to that in the control group (Figure 2). To determine whether the predicted miR-34c target sites in the 3'-UTRs of its target mRNAs were responsible for the silencing of gene expression by miR-34c, we cloned the putative 3'-UTR target sites downstream of a luciferase reporter gene and cotransfected this vector into 293T cells with either pre-miR-34c or a control precursor. In cells transfected with pre-miR-34c and pRl-ACSL4-WT, pRl-SAR1A-WT, pRl-PDE7B-WT, pRl-PDGFRA-WT, or pRL-MAP2K1-WT, luciferase activity was decreased relative to that in cells cotransfected with control precursor and mutated versions of the target (Figure 3). These results demonstrate that miR-34c can directly target seed sequences in the 3'-UTRs of target genes to suppress gene expression. In addition, we tested the expression level of miR-34c and its targets in pancreatic tissue to analyze the role of miR-34c and its targets in maintaining the normal physiological function of beta cells. *In situ* hybridization and IHC were used to measure the expression of insulin, miR-34c, and its targets in murine embryonic pancreatic tissue sections. The results demonstrated that insulin is co-expressed with ACSL4 and SAR1A in pancreatic islets, but not with miR-34c, MAP2K1, PDE7B, and PDGFRA (Figure 4). The results suggested that miR-34c plays a critical role in maintaining the normal physiological function of beta cells.

### Role of miR-34c and its targets of proinsulin synthesis and insulin secretion

**Figure 5:**
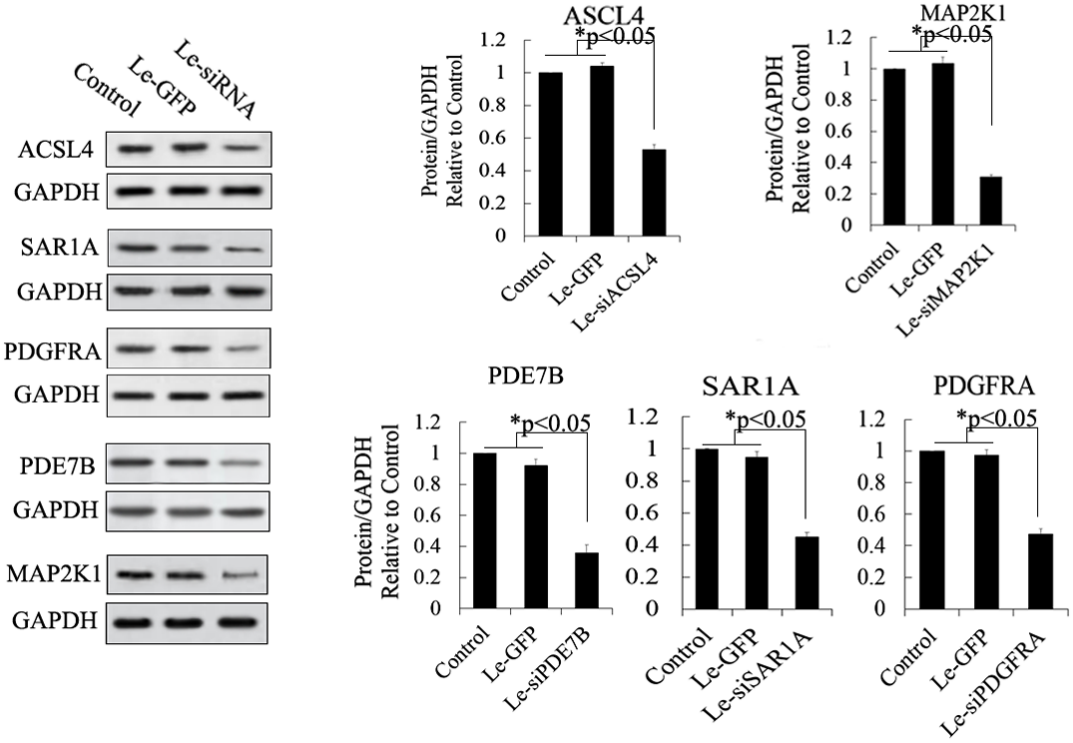
Western blot analysis of proteins expressed by siRNA-targeted genes in MSCs Protein abundance was analyzed using ImageJ tools. Overexpression of siRNA for 72 h inhibited the endogenous expression of ACSL4, PDE7B, PDGFRA, MAP2K1, and SAR1A (n = 5; paired two-tailed *t*-test, ^*^: p<0.05, ^**^: p<0.01). GAPDH was used as an endogenous control. Le-eGFP, lentivirus expressing enhanced green fluorescent protein (vector control); Le-siRNA, lentivirus expressing siRNA.

**Figure 6:**
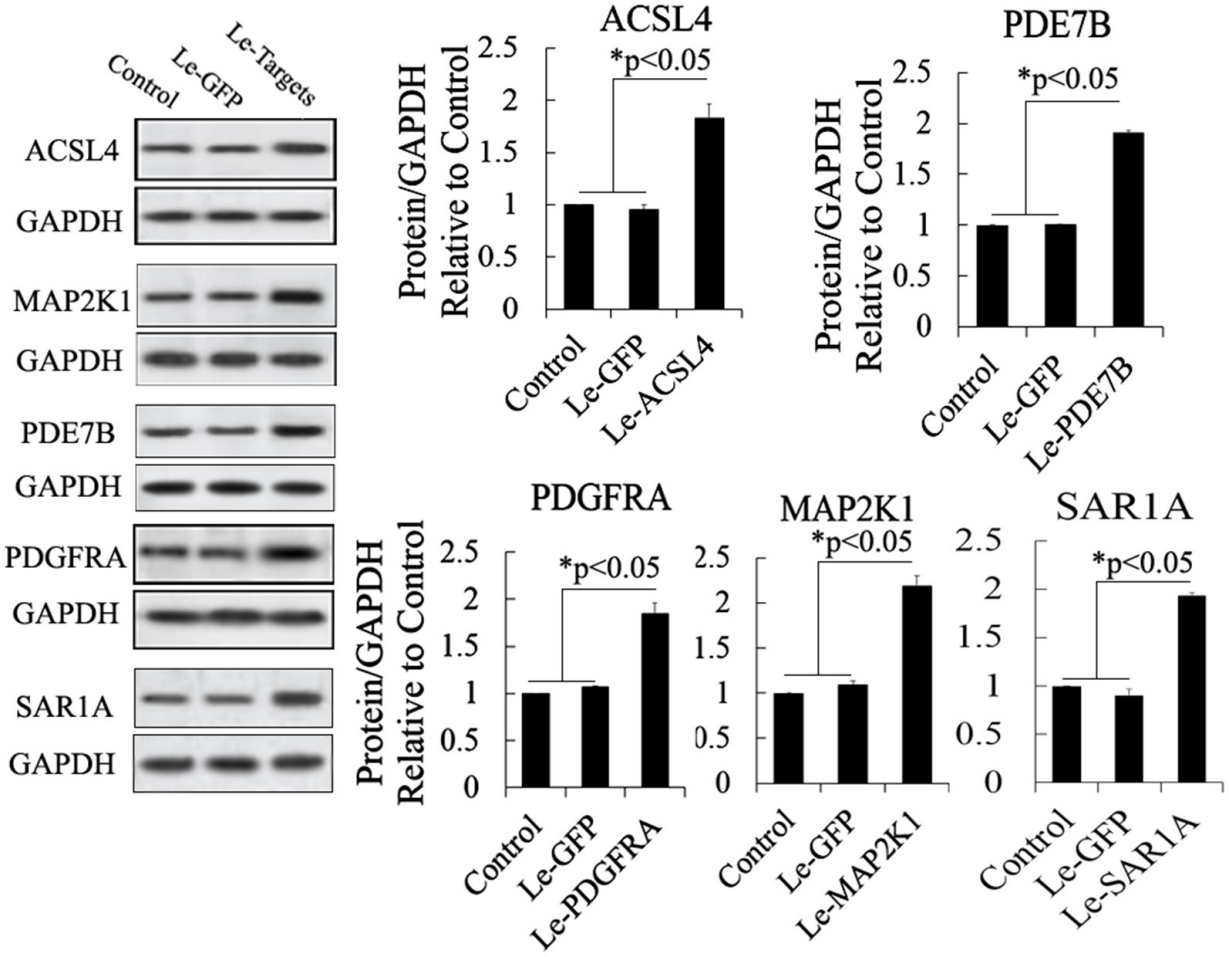
Western blot analysis of proteins expressed by gene targets of miR-34c following its mRNA overexpression in MSCs Protein abundance was analyzed using ImageJ tools. Overexpression of mRNA of putative target genes for 72 h increased the endogenous expression of ACSL4, PDE7B, PDGFRA, MAP2K1, and SAR1A (n = 5; paired two-tailed *t*-test, ^*^: p<0.05, ^**^: p<0.01). GAPDH was used as an endogenous control. Le-eGFP, lentivirus expressing enhanced green fluorescent protein (vector control); Le-Targets, lentivirus expressing putative target genes.

To investigate the function of miR-34c and its targets in the differentiation of IPCs derived from MSCs, we designed and constructed siRNA and an expression vector of targets of miR-34c to research its role in the differentiation of IPCs, and these vectors can effectively modulate the expression of targets of miR-34c, as shown in Figures 5 and 6. For example, ACSL4, as a target of miR-34c, plays an important role in insulin secretion in pancreatic beta cells, participating in lipid metabolism to enhance insulin synthesis upon glucose-stimulated insulin secretion [26, 33]. ACSL4 was downregulated by the overexpression of miR-34c in PSCs. Other targets include MAP2K1, also known as MEK, which plays an important role in cell proliferation, and PDE7B, which functions in the exocytosis of beta cells and alpha cells of the pancreas. Overexpression of PDE7B was reported to perturb insulin and glucagon secretion in these cells [27]. PDGFRA is a “disallowed” gene in pancreatic beta cells; its overexpression in beta cells when glucose induced insulin secretion caused cell dysfunction to inhibit insulin secretion [32]. A further target of miR-34c is SAR1A, which plays an important role in the endoplasmic reticulum (ER) control of insulin secretion. Defective Sar1 was reported to block proinsulin ER export and to abolish its conversion to mature insulin in MIN6 cells [29, 30]. In our research, bioinformatic analysis showed that the 3′-UTR of these genes has a binding site of miR-34c, and luciferase report assay and western blotting also demonstrated that miR-34c directly regulates its targets. Meanwhile, these targets of miR-34c also revealed significant changes in expression during the differentiation of IPCs derived from MSCs, and the above results suggest that miR-34c and its targets may be specifically involved in the differentiation of IPCs.

**Figure 7:**
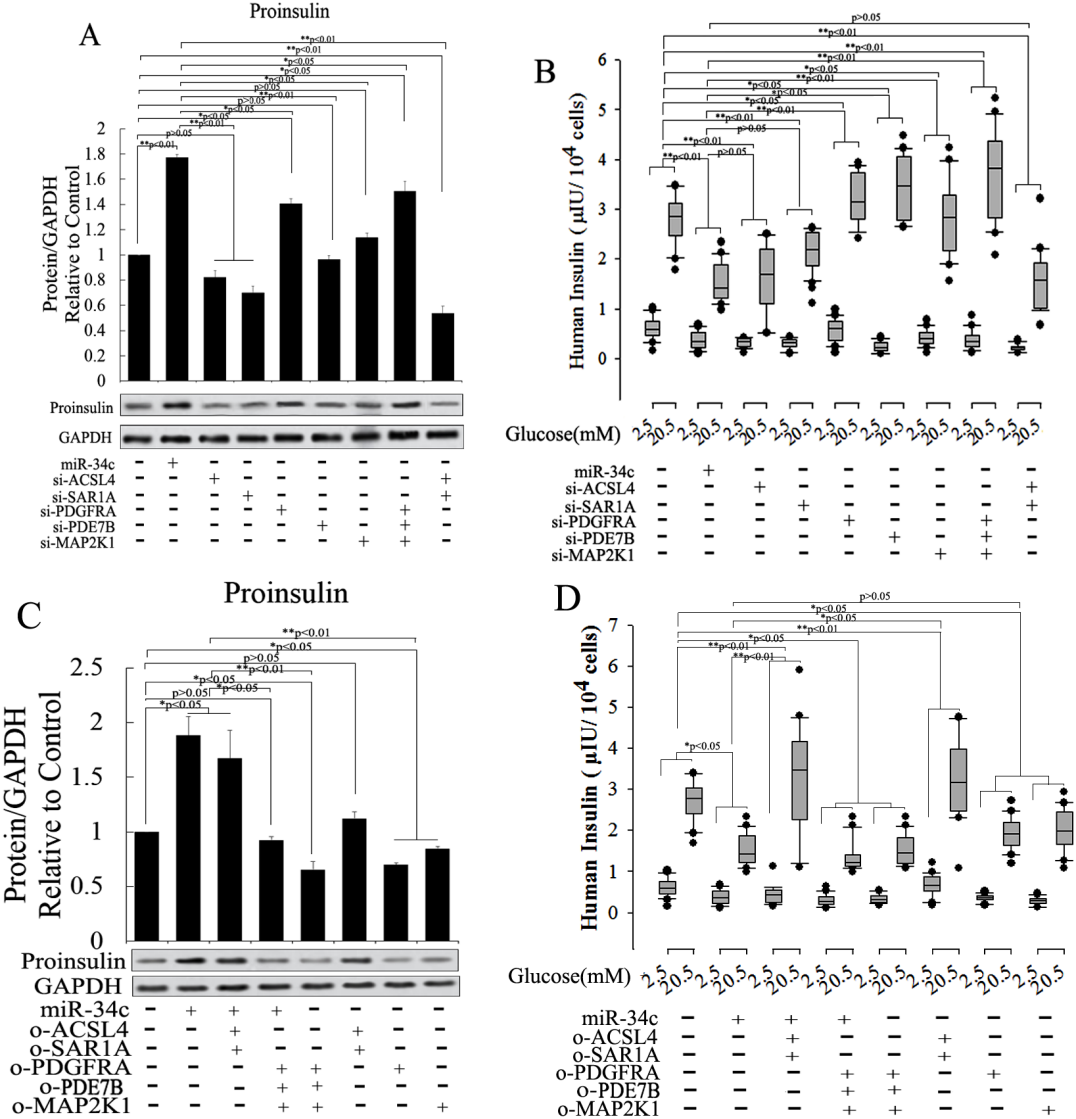
Role of miR-34c and its targets of proinsulin synthesis and insulin secretion To investigate the function of miR-34c and its targets in the differentiation of IPCs derived from MSCs, we designed and constructed siRNA and expression vector of targets of miR-34c to research its role in the differentiation of IPCs. **(A** and **B)** Single expression of miR-34c, si-PDGFRA, si-PDE7B, and si-MAP2K1 in IPCs showed that these small nucleotides could increase intracellular proinsulin synthesis; meanwhile, when si-PDGFRA, si-PDE7B, and si-MAP2K1 were expressed singly or in combination in IPCs, the IPCs derived from MSCs also promoted insulin secretion, but the expression of miR-34c decreased insulin secretion under treatment with 20.5 mM glucose. When si-ACSL4 and si-SAR1A were expressed singly or in combination in MSCs, the IPCs derived from these cells not only inhibited intracellular proinsulin synthesis, but also decreased insulin secretion after treatment with 20.5 mM glucose. **(C** and **D)** Overexpression of miR-34c and its targets to investigate its function in insulin synthesis and secretion. Overexpression of miR-34c and its targets (ACSL4 and SAR1A) not only increased intracellular proinsulin synthesis, but also promoted insulin secretion; upon the combined overexpression of miR-34c, MAP2K1, PDE7B, and PDGFRA in MSCs, we found that these genes inhibited intracellular proinsulin synthesis and insulin secretion. Single gene expression revealed that MAP2K1, PDE7B, and PDGFRA act as inhibitors of proinsulin synthesis and insulin secretion, while ACSL-4 and SAR1A act as promoters of proinsulin synthesis and insulin secretion during the differentiation of IPCs (^*^: p<0.05, ^**^: p<0.01).

**Figure 8:**
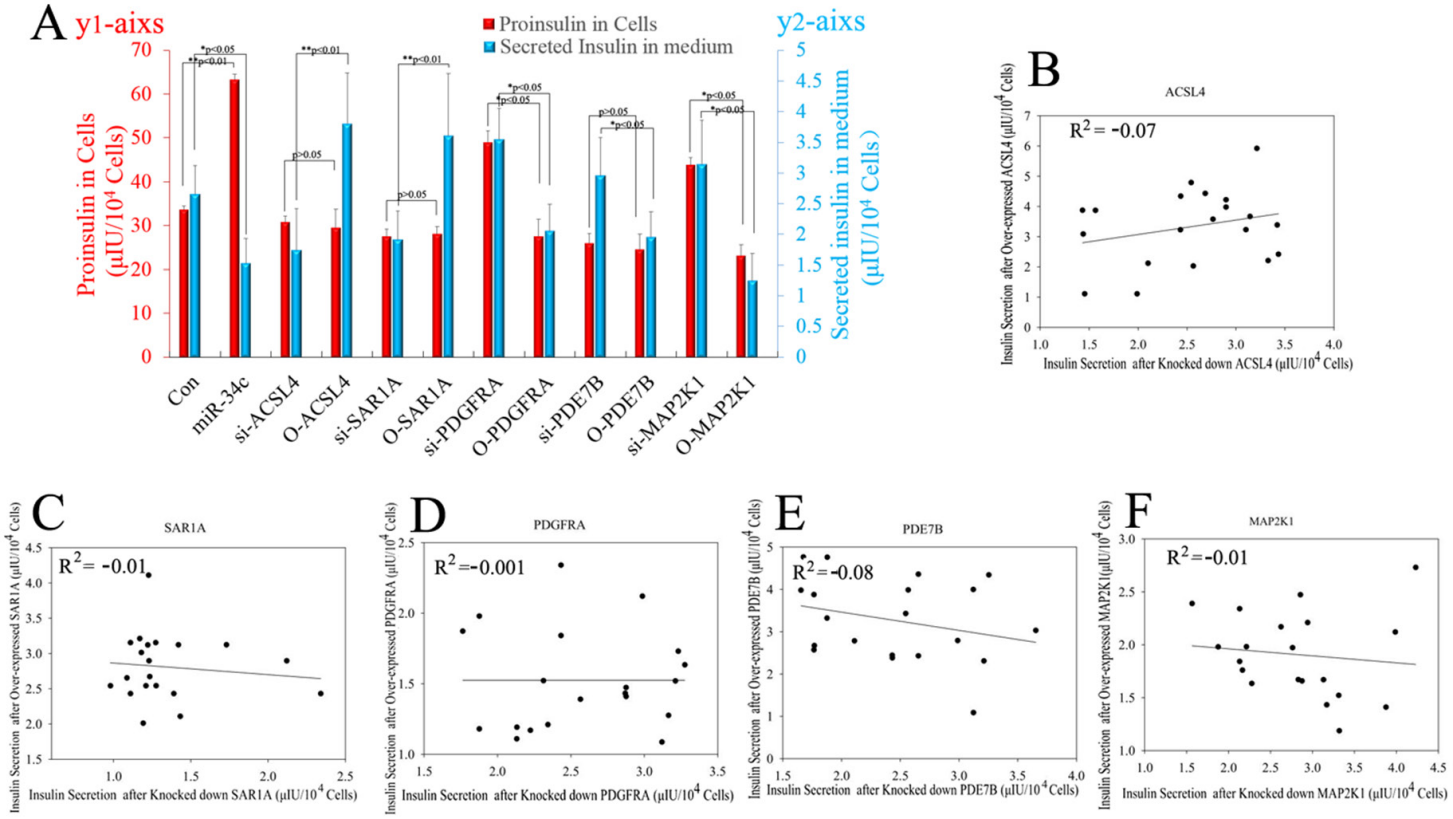
Statistical analysis of miR-34c and its targets affecting proinsulin synthesis and insulin secretion **(A)** miR-34c only increased intracellular proinsulin synthesis compared with that in the control group in IPCs derived from MSCs (P<0.01), but weakened insulin secretion under high-glucose treatment (P<0.05). These results indicated that miR-34c targets play a critical role in proinsulin synthesis and insulin secretion: MAP2K1, PDE7B, and PDGFRA are disordered genes, and ACSL4 and SAR1A are regulatory genes necessary for proinsulin synthesis and insulin secretion. Proinsulin in cells corresponds to the red Y-axis (left); secreted insulin in medium corresponds to the blue Y-axis (right). **(B–F)** Relevant analysis of miR-34c targets in regulated insulin secretion showed negative correlation between ectopic expression and knockdown in IPCs. These results demonstrated that miR-34c targets play an important role in the function of IPCs (^*^: p<0.05, ^**^: p<0.01).

In another experiment about the role of miR-34c and its targets in insulin synthesis and secretion, miR-34c, siRNA, and mRNA of its targets were expressed in MSCs singly or in combination and then these cells were induced to differentiate into IPCs. Next, the functions of IPCs after treatment with 20.5 mM glucose, including proinsulin synthesis and insulin secretion, were tested, with the results shown in Figure 7. Single expression of miR-34c, si-PDGFRA, si-PDE7B, and si-MAP2K1 in IPCs showed that these small molecules could increase intracellular proinsulin synthesis. Meanwhile, when si-PDGFRA, si-PDE7B, and si-MAP2K1 were expressed singly or in combination in MSCs, the IPCs derived from these MSCs also exhibited the promotion of insulin secretion, but the expression of miR-34c decreased insulin secretion under treatment with 20.5 mM glucose. Moreover, when si-ACSL4 and si-SAR1A were expressed singly or in combination in MSCs, the IPCs derived from these cells not only inhibited intracellular proinsulin synthesis, but also decreased insulin secretion after treatment with 20.5 mM glucose (Figure 7A and 7B). Based on the above results, we asserted that the overexpression of miR-34c and its targets singly or in combination in MSCs had adverse effects. Overexpression of miR-34c and its targets (including ACSL4 and SAR1A) not only increased intracellular proinsulin synthesis, but also promoted insulin secretion; in addition, upon combined overexpression of miR-34c, MAP2K1, PDE7B, and PDGFRA in MSCs, we found that these genes inhibited intracellular proinsulin synthesis and insulin secretion. Moreover, single gene expression revealed that MAP2K1, PDE7B, and PDGFRA act as inhibitors of proinsulin synthesis and insulin secretion, while ACSL-4 and SAR1A act as promoters of these processes during the differentiation of IPCs (Figure 7C and 7D). Taking these findings together, miR-34c alone increased intracellular proinsulin synthesis compared with that in the control group in IPCs derived from MSCs (*p*<0.01), but weakened insulin secretion under high-glucose treatment (P<0.05). These results indicate that miR-34c targets play a critical role in proinsulin synthesis and insulin secretion: MAP2K1, PDE7B, and PDGFRA as disordered genes, and ACSL4 and SAR1A as regulatory genes necessary for proinsulin synthesis and insulin secretion (Figure 8A). Relevant analysis of these genes in regulated insulin secretion showed negative correlation between its ectopic expression and knockdown in IPCs. These results demonstrate that miR-34c targets play a critical role in the function of IPCs (Figure 8B–8E).

## DISCUSSION

miRNAs are endogenous, small, noncoding ribonucleotides present in animals and plants. They play important roles in the regulation of gene expression at the post-transcriptional level [9, 34, 35]. Only limited studies have been performed on the expression and function of miRNAs in the differentiation of insulin-producing cells from MSCs. The replacement of IPCs would be the ideal solution for the treatment of diabetes mellitus; however, the generation of insulin-secreting cells from stem cells represents an attractive alternative. In this study, we found that miR-34c was transiently upregulated during the formation of IPCs derived from MSCs, when we verified the results of RNA-seq using qPCR. To the best of our knowledge, there are no reports about miR-34c’s involvement in the function and maturation of pancreatic beta cells, so we analyzed targets of miR-34c using bioinformatic tools. These genes include a combined site of miR-34c in the 3’-UTR, which plays an important role in insulin synthesis and secretion of pancreatic beta cells, and include ACSL4, SAR1A, PDE7B, PDGFRA, and MAP2K1.

Pancreatic beta cells possess highly active protein synthetic and export machinery in the ER to accommodate the massive production of proinsulin. ER homeostasis is vital for beta-cell functions and is maintained by a delicate balance among protein synthesis, folding, export, and degradation. Secretion-associated Ras-related GTPase 1A (SAR1A) is known to specifically block coat protein complex II (COPII)-dependent ER export. Small interfering RNA-mediated knockdown of Sar1 demonstrated that defective Sar1 function blocked proinsulin ER export and abolished its conversion to mature insulin in beta cells and primary cultured islets [29]. Long-chain acyl-CoA synthetases (ACSLs) convert fatty acids to fatty acyl-CoAs to regulate various physiological processes, the isoforms of which include ACSL1, and ACSL3-ACSL6. ACSL3 and ACSL4 proteins were found to be present in beta cells and pancreatic islets and to be concentrated in insulin secretory granules, less so in mitochondria, and present at negligible levels in other intracellular organelles [33]. In this study, SAR1A and ACSL4 were targets of miR-34c, and knockdown of SAR1A and ACSL4 caused the decline of insulin secretion in IPCs derived from MSCs, suggesting that miR-34c could play a critical role in the insulin secretion of IPCs derived from MSCs by targeting SAR1A and ACSL4.

PDGFRA has recently been revealed to be one of the “disallowed” genes, which despite being strongly expressed and playing housekeeping roles in most other mammalian tissues, is expressed at an unusually low level in pancreatic beta cells. The overexpression of PDGFRA in beta cells was found to contribute to the dysfunction of insulin secretion, although the mechanisms involved in this dysfunction remain poorly understood. Aida et al. revealed a role for miRNAs in the regulation of disallowed genes in beta cells and provided evidence for a novel means through which noncoding RNAs control the functional identity of these cells independently of actions on beta cell mass [31]. PDE7B is a cAMP-specific phosphodiesterase that regulates cellular cAMP levels [36]. Although this is the first study showing that PDE7B affects insulin secretion, other members of this family are known to control cAMP and insulin secretion in beta cells [37]. Dayeh et al. demonstrated that the overexpression of PDE7B resulted in decreased glucose-stimulated insulin secretion in clonal beta cells [27]. In agreement with these previous findings, our research showed that PDE7B mRNA is significantly downregulated after differentiation [27]. MAP2K1, also known as MEK1, plays an important role in the MAPK signaling pathway and takes part in the regulation of cell proliferation, being located upstream of extracellular-regulated kinase (ERK) and acting as a promoter in its activation. However, there are no reports about MEK-regulated expression of an insulin gene, but MEK can affect the expression of CCND1. CCND1 is a major member of the Cyclin D family, essential for pancreatic beta-cell growth. CCND1 is also a downstream gene of the MAPK signaling pathway, and its expression must be downregulated and a low expression level must be maintained during the insulin synthesis phase of beta cells [7, 38]. These results suggest that MEK1 can influence the expression level of CCND1, and downregulated MEK1 expression may promote insulin synthesis and secretion. In the current study, the expression of PDGFRA, PDE7B, and MAP2K1 was significantly downregulated during the differentiation of IPCs derived from MSCs induced by cocktail factors. Meanwhile, these genes were targets of miR-34c, which can post-transcriptionally regulate the level of target gene expression to increase proinsulin synthesis and insulin secretion in IPCs derived from MSCs.

In conclusion, we used the overexpression of miR-34c and specific siRNAs to elucidate the role of miR-34c and its targets in the maturation of IPCs derived from MSCs, with these targets including ACSL4, SARA1A, PDE7B, PDGFRA, and MAP2K1. PDE7B regulates cellular cAMP levels to affect insulin secretion in beta cells, PDGFRA is a “disallowed” gene for maintaining cell function in beta cells, and MAP2K1 plays a key role in cell proliferation, regulating CCND1 expression to disturb insulin secretion. These targets of miR-34c were negative regulators of IPC differentiation and insulin synthesis and secretion, and transient upregulation of miR-34c at an early stage of IPC differentiation suggested that miR-34c downregulates the above targets to promote PC differentiation. However, ACSL4 regulates the metabolism of fatty acids to increase insulin secretion, and SAR1A plays an important role in endoplasmic reticulum-mediated production of proinsulin. A low level of miR-34c is beneficial at the later stage of PC differentiation for producing normal physiological function. Therefore, miR-34c acts as a bidirectional switch in the formation of IPCs by regulating the expression of target genes. Additional studies focusing on specific miRNAs involved in the formation of IPCs from stem cells may advance the development of effective cell transplant therapies for diabetes mellitus.

## MATERIALS AND METHODS

### MSC culture and directional differentiation *in vitro*

Wharton’s jelly was obtained from mouse embryos and then digested with collagenase type IV (Sigma, USA) for the isolation of MSCs. MSCs were cultured in L-DMEM (Gibco, USA) supplemented with 10% (v/v) fetal bovine serum (FBS; Biochrom, Germany). Markers of MSCs, including CD44, CD71, CD73, CD90, CD105, and Nestin, were analyzed using flow cytometry. For the differentiation of insulin-producing cells, the cocktail factor method was used in this research; in brief, MSCs were exposed to 1 μM 5-AZA for 18 h before induction. Then, they were cultured in low-glucose DMEM and 10% fetal bovine serum (FBS) supplemented with 0.1 mM β-mercaptoethanol (Sigma) and 1 mM nonessential amino acids (Gibco). On day 6, the medium was supplemented with 15 ng/mL activin A (Sigma). On day 7, 10 mM retinoic acid (Sigma) was added. Two days later, medium was replaced with medium supplemented with 1% B27 (Gibco), 10 ng/mL basic fibroblast growth factor (Peprotech, USA), 10 mM nicotinamide, and 1% ITS (insulin, transferrin, selenium solution; Gibco) to encourage further differentiation.

### Glucose-stimulated insulin secretion

Insulin-producing cells were washed with Krebs buffer and then preincubated in low-glucose (2.5 mM) Krebs buffer for 2 h to remove residual insulin. Beta cells were washed three times in Krebs buffer, incubated in low-glucose Krebs buffer for 30 min, and then the supernatant was collected. Next, beta cells were washed three times in Krebs buffer and incubated in high-glucose (20.5 mM) Krebs buffer for 30 min, after which the supernatant was collected. This sequence was repeated three additional times. Finally, clusters were incubated in Krebs buffer containing 2.5 mM glucose. Supernatant samples containing secreted insulin were processed using an insulin ELISA test.

### Small RNA library construction and sequencing

The Illumina TruSeq Small RNA Sample Preparation protocol was used to prepare RNAs. In brief, total RNA samples were size-fractionated by 15% PAGE, and the 16–40-nt fractions were collected. The 5′ and 3′ RNA adaptors were ligated to the RNA, and RNAs of 64–99 nt were isolated by gel elution and ethanol precipitation. Polymerase chain reaction (PCR) products were purified and small RNA libraries were sequenced using the Illumina Genome Analyzer (Illumina Genome Analyzer_IIX_). The raw data were processed using Illumina Genome Analyzer Pipeline software and then submitted for data filtration. Clean reads were obtained after filtering low-quality reads and trimming the adaptor sequences. All of the clean reads were initially searched against miRBase (version 21; http://www.mirbase.org/) to identify miRNAs. Unmappable reads were annotated and classified by reference to noncoding RNAs in the Ensembl (http://http://ftp.ensembl.org/pub/release-69/fasta/sus_scrofa/ncrna/), piRNA (http://pirnabank.ibab.ac.in/), and Rfam (version 10; http://rfam.sanger.ac.uk/) databases. The mappable sequences were used for further analysis. Meanwhile, many unannotated sequences for which no match was found in any of the above databases were analyzed by miRDeep (http://deepbase.sysu.edu.cn/miRDeep.php) to predict novel miRNA candidates. After all annotation steps, the sequencing libraries were used for size distribution and saturation analyses. miRNA expression was compared between MSCs and IPCs to determine which miRNAs were differentially expressed. The expression levels of miRNAs within each library were normalized to obtain the expression in transcripts per million (Tpm) mapped reads. R package DEGseq software was used to identify differentially expressed miRNAs. *p*-values for differentially expressed miRNAs were calculated using the MA-plot-based random sampling model (MARS). P-values were adjusted using q-value [20]. q-Value <0.01 and |log2(fold change)| >1 were set as thresholds for significant differential expression by default. Computational prediction of miRNA targets was performed in the online databases miRDB (http://www.miRdb.org/), miRanda (http://www.miRanda-im.org/), miRwalk (http://www.ma.uni-heidelberg.de/apps/zmf/miRwalk/), and TargetScan (http://www.targetscan.org/).

### Real-time PCR

Total RNA, including miRNAs, was isolated from MSCs using TRIzol reagent (Invitrogen, USA). cDNA synthesis was carried out with the High Capacity cDNA synthesis kit (Applied Biosystems, USA) using 2 ng of total RNA as a template. The sequence-specific reverse-transcription PCR primers for miRNAs and endogenous control U6 were synthesized by Sangon (Shanghai, China). Real-time PCR analysis was carried out using the Applied Biosystems 7500 real-time PCR system. The gene expression cycle threshold (CT) values of miRNAs from each sample were calculated by normalization to those of internal control U6 and relative values were plotted.

### Venn diagrams

Venn Tools was used to demonstrate superposition relationships between target genes of miRNAs and microarray results. Venn diagrams were generated using software at http://bioinfogp.cnb.csic.es/tools/venny/index.html.

### Construction of lentiviral vectors

Precursor sequences for miRNAs and small interfering RNAs (siRNAs) were synthesized and cloned into a lentiviral vector. The vector was then incorporated into a lentivirus in HEK-293T cells. Viral particles were used to infect MSCs. The lentivirus also expressed green fluorescent protein as a marker of infection efficiency. Western blot analysis was used to detect the expression of the protein products of genes targeted by miRNAs and siRNAs.

### Western blotting

ACSL4, SAR1A, PDE7B, PDGFRA, and MAP2K1 were detected by western blot analysis following the overexpression of miR-34c or siRNA. Cells were lysed using M-PER Protein Extraction Reagent (Pierce, USA) supplemented with a protease inhibitor (PMSF). Protein concentrations of the extracts were measured with the BCA assay (Pierce, USA) and equalized with extraction reagent. Equal amounts of extracts were loaded and subjected to SDS-PAGE, followed by transfer onto nitrocellulose membranes. Primary antibodies (ACSL4, 1:500; SAR1A, 1:500; PDE7B, 1:800; PDGFRA, 1:500; and MAP2K1, 1:200) and horseradish peroxidase-coupled secondary antibodies (1:2000) were purchased from Abcam, USA. Membranes were probed using ultra-enhanced chemiluminescence western blotting detection reagents. GAPDH was used as an internal control. Protein abundance was analyzed using ImageJ tools.

### Flow cytometry

To determine the levels of Nestin and proinsulin expression, we analyzed cells with a Beckman Coulter FC500 flow cytometer. Briefly, cells were detached with 0.125% trypsin, centrifuged, separated into 500-μL aliquots, and labeled with custom-made FITC-conjugated polyclonal antibodies against Nestin or proinsulin (MBL International, Japan) as per the manufacturer’s instructions [21]. Flow cytometric data were analyzed using CXP software (Beckman Coulter, USA). Mean fluorescence intensity was determined after subtraction of a respective negative control.

### Luciferase reporter assay

Firefly luciferase reporter genes were constructed using the pCS2-Luc vector and the 3' UTRs of targets of miR-34c. Primers for PCR amplification of the 3' UTRs were as follows: *PDE7B*, 5'-GAATTC TGTGTGTAGTAAGCTGACGGT-3' (forward) and 5'-CTCGAG AGGTCCTGACAATTCATCACTGT-3' (reverse); *ACSL4*, 5'-GAATTC CCGCCAACTCCCAAAAGCTA-3' (forward) and 5'-CTCGAG AGAATTAGCAGCACCCAACCT-3' (reverse); *MAP2K1*, 5'-GAATTC AGAACTCAGCAGTTGACATCC-3' (forward) and 5'-CTCGAG GTAGACAGACTGGTGAAGCCC-3' (reverse); *PDGFRA*, 5'-GAATTC CCAGTACTGACTTGTGGGGAAA-3' (forward) and 5'-CTCGAG ATACTTCACTGAGCCTCTGC-3' (reverse); and *SAR1A*, 5'-GAATTC CTCCAGTACTGACTTGTGGGG-3' (forward) and 5'-CTCGAG ACTTCACTGAGCCTCTGCAT-3' (reverse). The underlined sequences indicate introduced *Eco*RI and *Xho*I sites. Constructs containing a mutated *PDE7B* (*PDE7B*-MUT), ACSL4 (ACSL4-MUT), PDE7B (PDE7B-MUT), PDGFRA (PDGFRA-MUT), or MAP2K1 (MAP2K1-MUT) 3' UTR were used as negative controls. Mutations at positions 3–5 of the miR-34c seed sequence were introduced using the QuikChange mutagenesis kit (Stratagene, USA). 293T cells were cultured in H-DMEM supplemented with 10% FBS. The cells were seeded into 24-well plates (2 × 10^4^ cells/well). After 24 h of culture, the cells were transfected using Lipofectamine 3000 (Invitrogen, USA) with a mixture containing 1 mg/mL firefly luciferase reporter plasmid, 20 nM miR-34c or control precursor, and 20 ng/mL of a plasmid encoding *Renilla reniformis* luciferase (pRL-TK; Promega, USA). Cells transfected without the precursor served as controls for normalization. Luciferase activity was measured 48 h after transfection using a dual-luciferase assay system (Promega). All transfections were repeated independently at least three times.

### *In situ* hybridization

Pancreatic tissue from embryos was fixed in 4% paraformaldehyde (PFA) in phosphate-buffered saline (PBS) overnight at 4°C and then treated with proteinase K for 30 min. *In situ* hybridization was performed as previously described [22]. Locked nucleic acid (LNA) probes were designed and synthesized by Sangon Biotech. The sequence of the LNA probe complementary to mature miR-34c was TCCGTCACATCAATCGACTAACG. LNA probes were labeled with digoxigenin using the DIG Oligonucleotide 3'-end Labeling Kit (Roche, USA) and purified using Sephadex G-25 MicroSpin columns (Amersham, Sweden). Images of pancreatic tissue were taken using a confocal microscope with a digital acquisition system (T-2000; Nikon, Japan).

### Immunohistochemistry

The methods used for immunohistochemistry (IHC) were as previously described [23]. Pancreas samples were dissected from mice and fixed in a 4% PFA/PBS solution overnight and embedded in paraffin. Five-micrometer longitudinal sections cut from paraffin blocks were rehydrated with xylene followed by decreasing concentrations of ethanol, microwaved in 0.01 M sodium citrate (pH 6.0) for 20 min, and permeabilized with 1% Triton X-100 in PBS prior to incubation overnight with primary antibody at 4°C. Primary antibodies against insulin, ACSL4, SAR1A, PDE7B, PDGFRA, and MAP2K1 were used at dilutions of 1:200, 1:100, 1:200, 1:100, 1:100, and 1:50, respectively. Sections were then incubated with GARB (1:400) and GAMB (1:100) for 3 h at room temperature and then with FITC or Cy3-conjugated streptavidin (1:50) for 3 h at room temperature. After counterstaining with 4′, 6-diamidino-2-phenylindole (DAPI), images were obtained by confocal microscopy.

### Statistical analysis

Data are expressed as the mean ± SD. Differences between experimental groups were assessed using the two-tailed t-test. Statistical significance was defined as ^*^P < 0.05 and ^**^P < 0.01.

## SUPPLEMENTARY MATERIALS TABLES








