# Supplementary material for: MicroRNA-34c acts as a bidirectional switch in the maturation of insulin-producing cells derived from mesenchymal stem cells
Source: Oncotarget. 2017 Oct 16;8(63):106844–57. doi: 10.18632/oncotarget.21883 (PMC5739778; doi:10.18632/oncotarget.21883)
Supplement: Supplementary file 1 [file oncotarget-08-106844-s001.pdf]

MicroRNA-34c acts as a bidirectional switch in the maturation of insulin-producing cells derived from mesenchymal stem cells

SUPPLEMENTARY MATERIALS

Supplementary Table 1: The differential expression of miRNAs during the differentiation of IPCs from MSCs

See Supplementary File 1

Supplementary Table 2: Predicted targets of miR-34c using bioinformatic tools

See Supplementary File 2

Supplementary Table 3: Putative target genes of miR-34c with regarded to pancreatic development

See Supplementary File 3
